# Multiscale Analysis Reveals Altered Characteristics in Femur and Mandible of Mice on a High Phosphate Diet

**DOI:** 10.1007/s00223-025-01425-2

**Published:** 2025-08-27

**Authors:** Kristin Nguyen, Minji Kim, Andrew J. Cheline, Peter Tsatalis, Yasaman Samanian, Olivia Jackson, Daniel A. Branch, Hannah F. Sanders, Farah A. Al-Omari, Young C. Jang, Beth S. Lee, Kedryn K. Baskin, Do-Gyoon Kim

**Affiliations:** 1 Division of Orthodontics, College of Dentistry, The Ohio State University, Columbus, OH 43210 USA; 2 Department of Physiology and Cell Biology, College of Medicine, The Ohio State University, Columbus, OH 43210 USA; 3 Dorothy M. Davis Heart and Lung Research Institute, The Ohio State University Wexner Medical Center, Columbus, OH 43210 USA; 4 Department of Orthopedics, Emory Musculoskeletal Institute, Emory School of Medicine, Atlanta, GA 30329 USA

**Keywords:** High phosphate diet, Bone, Mineral Metabolism, Microcomputed tomography, Mineral density, Morphology, Mechanical stability, Dynamic mechanical analysis

## Abstract

Excessive phosphate used as flavor enhancers and preservatives in processed foods can exacerbate cardiovascular and kidney diseases. In clinical and pre-clinical studies, chronic (over 52 weeks) high-phosphate diet (HPD) negatively affects bone health. We previously demonstrated that 12-week-HPD decreases exercise capacity and skeletal muscle metabolism in adult male mice; however, alteration of bone characteristics associated with HPD independent of disease complications is not well-characterized. Thus, we determined the effects of shorter-term-HPD on characteristics of mouse femurs and mandibles. Adult male mice were fed a normal phosphate diet (NPD) or HPD for 18 weeks, serum markers of mineral metabolism and bone formation and resorption were quantified in femurs, and histological analysis was performed on tibias. Volumetric, mineral density, and morphology parameters of femurs and mandibles were determined using micro-computed tomography, and dynamic mechanical analysis and fracture testing of the femur were conducted. Our studies revealed that 18-week-HPD significantly reduced bone quality (tissue mineral density (TMD) and cortical thickness) without changing bone quantity (total mineral content and volume) of both femurs and mandibles, and femur mechanical properties were aggravated increasing the risk of fracture. Serum markers of osteoclastic resorption and osteoblastic formation were increased with HPD, indicating active osteoclastic bone resorption and osteoblastic new bone formation. These findings provide detailed information on how excessive dietary phosphate substantially alters characteristics of bone, resulting in bone weakening.

## Introduction

Inorganic phosphate (Pi) is commonly used in excess as a flavor enhancer and preservative in processed foods [1]. Consequently, over 30% of the US population consumes more than twice the recommended dietary allowance of Pi on a regular basis [2]. In healthy adults, the phosphorus estimated average requirement (EAR) is 580 mg/day and the recommended dietary allowance (RDA) is 700 mg/day. However, the average phosphorus intake of US adults over the age of 20 is 1399 mg/day–approximately 2.5 times the EAR and twice the RDA [3]. Elevated serum Pi levels are not only associated with progression of cardiovascular and kidney diseases, but also with increased rates of mortality in otherwise healthy subjects [4].

Phosphate homeostasis is systemically regulated through the intestine-kidney-bone axis, and is hormonally controlled [5]. Clinical studies reported that an increase in serum phosphate is associated with increased risk of bone fracture in adults [3, 6]. In adult female mice, 1 month of acute HPD containing a three-fold increase in phosphate content reduced femur BMD and bone mass [7], and 1 year (52–64 weeks) of chronic HPD containing a two-fold increase in phosphate content reduced femur BMD and bone mass in female and male mice [8, 9]. However, detailed characterization of bone and active bone remodeling after shorter-term HPD (18 weeks), mimicking 2.5 times the phosphate EAR, in adult male mice has not been fully investigated, nor have effects of HPD on jaw bone been assessed. While jaw bone is well-established to be sensitive to metabolic disruptions such as hyperparathyroidism and hypophosphatemic rickets [10, 11], at the same time differences in the embryonic origin of bone mesenchymal stem cells lead to greater osteogenic capacity in mandibles versus limb bones, thus resulting in less susceptibility to other conditions such as age-related osteoporosis [12, 13]. Therefore, comparison of HPD effects on both femur and mandible are necessary. Additionally, previous rodent studies in adult animals were limited in their methodologies and did not take advantage of more comprehensive multiscale technologies that include measurements such as tissue mineral density and mechanical testing [7–9].

While bone quantities such as mass and volume have been used as surrogates to estimate mechanical properties of bone, many studies suggest that bone qualities including tissue mineral density (TMD) and morphology also contribute to determining the mechanical behavior of bone [12, 14–16]. TMD is a measurement of mineral contents in bone tissue, excluding bone marrow and other pores. As the newly formed bone tissue has less mineral than existing bone tissues, TMD is heterogenous, reflecting the degree of bone tissue mineralization resulting from bone modeling and remodeling [17]. In previous studies, we found that the heterogeneity of TMD plays an important role in determining diverse mechanical characteristics of bone, including elastic, viscoelastic, and fracture behavior [12, 14]. We hypothesize that HPD triggers active bone modeling and remodeling to change the quantity and quality of bone, and increases the risk of bone fracture. Thus, the objective of the current study was to determine the effects of 18-week-HPD on characteristics of male mouse femurs and mandibles.

## Materials and Methods

### Mouse Studies

Following the approved protocol of the Ohio State University Institutional Animal Care and Use Committee (2018A00000121-R1), 18-week-old male C57BL/6 J mice were obtained from Jackson Laboratory (Bar Harbor, ME) and were acclimated to the new environment for 2 weeks on control (normal phosphate) diet (NPD) containing 0.6% Pi (9.2 g/kg) (TD160114, Envigo Teklad Diets, Madison, WI). After acclimation, 20-week-old mice were randomized into dietary treatment groups (n = 15/group) and fed either NPD or a diet containing high phosphate (HPD) (2.0% Pi, 22.8 g/kg; TD08020) for 18 weeks. These diet formulations have been used in numerous high phosphate diet studies in mice and mimic human phosphorus intake of 2.5 times the EAR and twice the RDA [18–20]. All other mineral contents of the diets were the same (0.3% magnesium, 1.9% calcium, 1.8% potassium, and 0.9% sodium). Animals were housed 2–3 per cage in a room-temperature and light-controlled room with a 12-h light/dark cycle. Food and water were provided ad libitum and mice were maintained in accordance with IACUC protocols.

### Physiological Measurements and Sample Collection

**Fig. 1:**
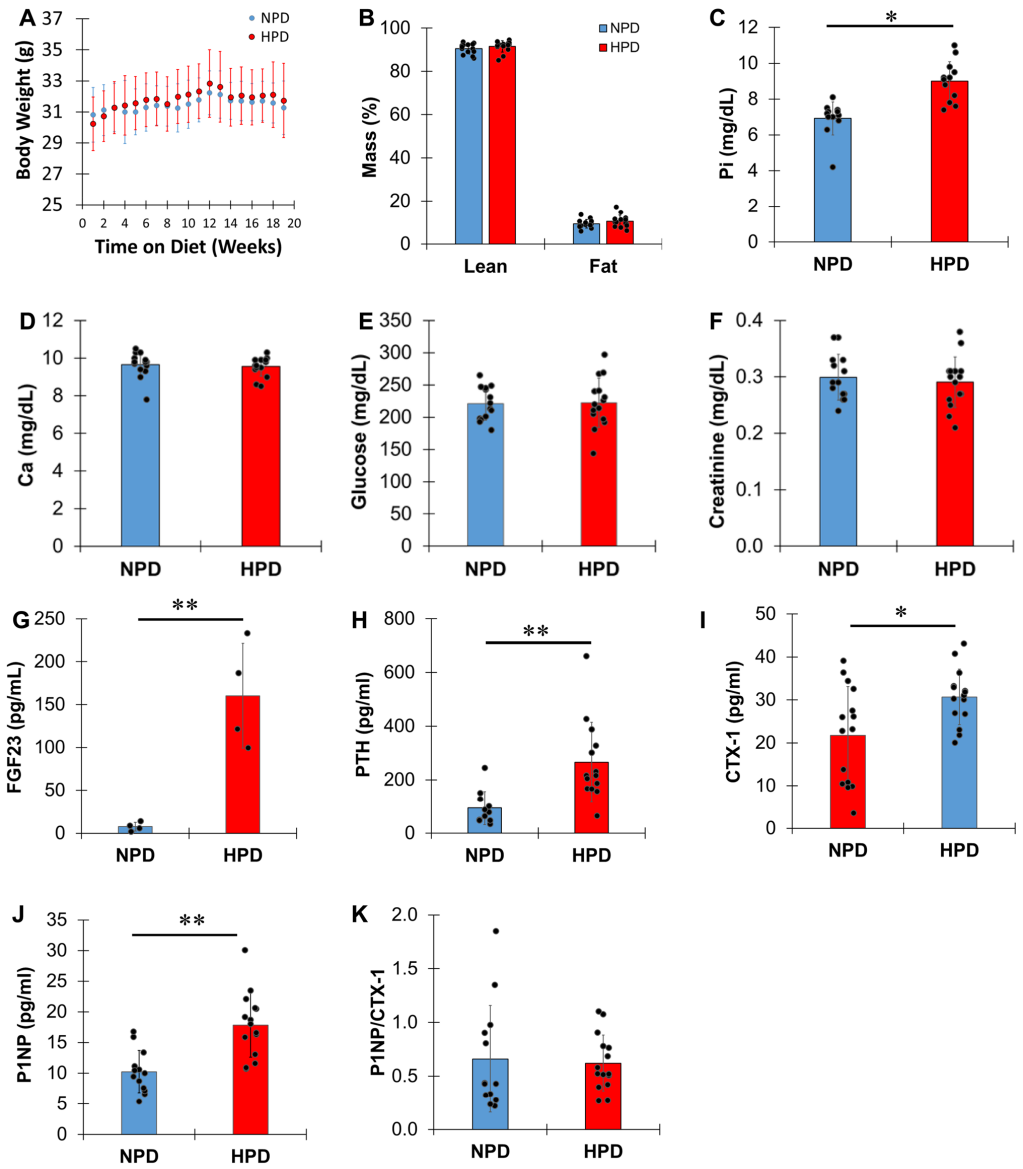
High phosphate diet increases serum phosphate levels and biomarkers of bone remodeling. **A** Body weight of male mice on NPD and HPD (n = 15 for each group) for 18 weeks. **B** Lean and fat mass of mice after 17 weeks on NPD and HPD. **C** Serum phosphate, **D** serum calcium, **E** serum glucose, **F** serum creatinine, **G** total FGF23 **H** PTH, **I** CTX-I, **J** P1NP, and **K** the ratio of P1NP to CTX-I after 18 weeks on NPD and HPD. *; p < 0.05, **; p < 0.01

Mice (n = 15/group) were weighed weekly throughout the dietary study (Fig. 1A). After 17 weeks on diet, body composition was determined using EchoMRI (EchoMRI™3-in-1). For blood and bone collection, mice were euthanized by cervical dislocation and exsanguination after 18 weeks on diet. Blood was collected by cardiac puncture and kept at room temperature for 30 min. Serum was separated by centrifugation (1000xg for 10 min at 4 ℃), aliquoted on ice, and stored at -80 ℃ until further analysis. Femurs, tibiae, and mandibles were dissected after removal of soft tissues and wrapped in normal saline-soaked gauze to store at -20 ℃ until use.

### Serum Analysis

Serum (from n = 15/group) was analyzed for phosphate, calcium, creatinine and glucose content by The Ohio State University College of Veterinary Medicine Clinical Pathology Services. Serum fibroblast growth factor 23 (FGF23) total levels were analyzed using the mouse FGF23 ELISA Kit (Abcam, Cambridge, UK) according to manufacturer instructions. Serum parathyroid hormone (PTH) levels were analyzed using the Mouse PTH 1–84 ELISA kit (Quidel, San Diego, CA, USA) according to manufacturer instructions. C-terminal telopeptides of type I collagen were obtained using RatLapsTM (CTX-I) enzyme immunoassay (EIA) kit (Immunodiagnostic Systems, Boldon, UK) according to manufacturer instructions. Serum P1NP (Procollagen Type 1 Intact N-terminal Propeptide) levels were analyzed using the Mouse P1NP EIA kit (BioVender, Ashville, NC, USA) according to manufacturer instructions.

### Micro-Computed Tomography (Micro-CT)

**Fig. 2:**
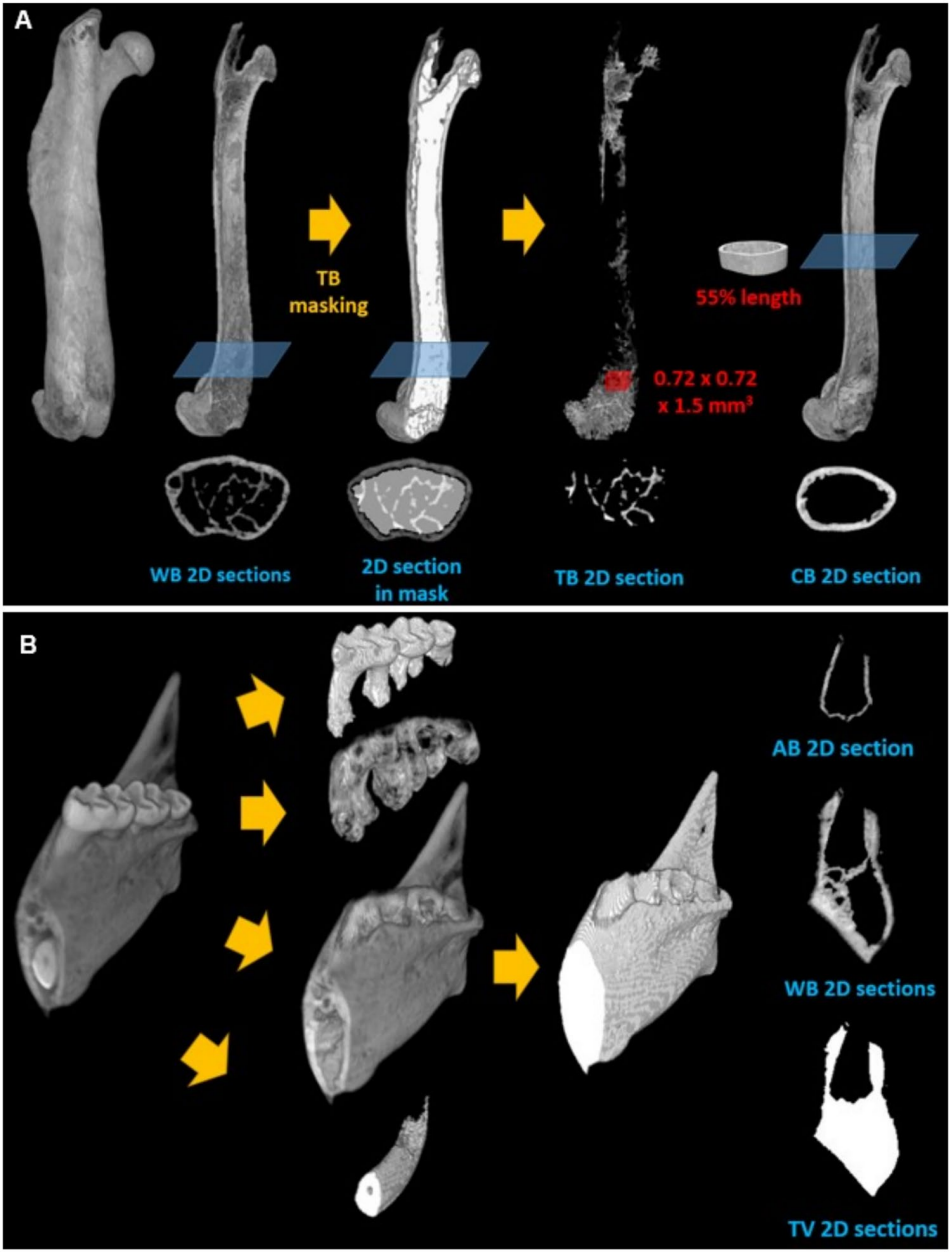
Femur and mandible analysis using 3D micro-computed tomography. Compartmentation of 3D micro-CT image to isolate **A** the cortical bone (CB) and trabecular bone (TB) from the whole bone (WB) of femur by masking the bone marrow cavity including TB, and **B** alveolar bone (AB) from the whole bone (WB) of mandible. A total volume (TV) of mandible was also assessed. Red cube and 55% length slice of femur in (A) indicates the regions of interest for TB and CB morphology, respectively

A femur and a mandible (n = 10 for each group) were thawed at a room temperature to be scanned by micro-CT (Skyscan 1172-D, Kontich, Belgium) with 20 × 20 × 20 µm^3^ voxel size (70 kVp, 141 μA, 0.4º rotation per projection, 6 frames averaged per projection, and 210 ms exposure time). The micro-CT images were analyzed following previous studies [12, 14]. In brief, volumetric parameters were obtained by counting bone-voxels that were segmented from non-bone voxels using a heuristic algorithm [21–23] (Fig. 2, Table 1,2).

**Table 1:** Comparison of measured parameters between normal phosphate diet (NPD) and high phosphate diet (HPD) groups (mean ± standard deviation) in the whole bone (WB), trabecular bone (TB), and cortical bone (CB) of femur. Significant (p < 0.05) or marginally significant (p < 0.07) differences are highlighted in bold

Parameters		NPD (n = 10)	HPD (n = 10)	p value
Volumetric	BV_WB_ (mm^3^)	25.75 ± 2.44	25.21 ± 1.47	0.55
	TV_WB_ (mm^3^)	46.89 ± 4.26	49.41 ± 3.77	0.18
	BV_TB_ (mm^3^)	4.88 ± 0.6	4.63 ± 0.73	0.42
	BV_CB_ (mm^3^)	21.36 ± 2.65	21.43 ± 1.86	0.95
Mineral Density	**BMD (mgHA/cm3)**	**865.26 ± 47.22**	**773.62 ± 43.89**	** < 0.001**
	TMC_WB_ (mgHA)	40.52 ± 3.83	38.12 ± 2.1	0.11
	TMC_TB_ (mgHA)	7.18 ± 0.99	6.47 ± 1.06	0.14
	TMC_CB_ (mgHA)	33.34 ± 3.03	31.65 ± 1.23	0.13
	**Mean**_**WB**_** (mgHA/cm**^**3**^**)**	**1574.32 ± 36.75**	**1512.75 ± 19.09**	** < 0.001**
	SD_WB_ (mgHA/cm^3^)	154.09 ± 8.16	153.12 ± 6.92	0.78
	**Low**_**5WB**_** (mgHA/cm**^**3**^**)**	**1319.32 ± 28.58**	**1267.17 ± 19.54**	** < 0.001**
	**High**_**5WB**_** (mgHA/cm**^**3**^**)**	**1810.5 ± 42.41**	**1758.92 ± 22.35**	**0.004**
	**Mean**_**TB**_** (mgHA/cm3)**	**1467.49 ± 30.37**	**1404.98 ± 20.37**	** < 0.001**
	SD_TB_ (mgHA/cm3)	124.54 ± 9.94	126.60 ± 5.93	0.58
	**Low**_**5TB**_** (mgHA/cm3)**	**1269.98 ± 30.32**	**1190.00 ± 32.02**	** < 0.001**
	**High**_**5TB**_** (mgHA/cm3)**	**1676.67 ± 42.98**	**1609.71 ± 14.32**	** < 0.001**
	**Mean**_**CB**_** (mgHA/cm**^**3**^**)**	**1599.41 ± 40.53**	**1536.52 ± 19.30**	** < 0.001**
	SD_CB_ (mgHA/cm^3^)	149.17 ± 8.58	148.15 ± 6.07	0.76
	**Low**_**5CB**_** (mgHA/cm**^**3**^**)**	**1340.30 ± 34.70**	**1294.81 ± 15.91**	**0.002**
	**High**_**5CB**_** (mgHA/cm**^**3**^**)**	**1818.01 ± 42.31**	**1768.60 ± 22.45**	**0.006**
Morphology	Ct.Ar (mm2)	0.73 ± 0.06	0.71 ± 0.11	0.85
	Tt.Ar (mm2)	1.76 ± 0.17	1.88 ± 0.18	0.16
	**Ct.Ar/Tt.Ar**	**0.41 ± 0.02**	**0.38 ± 0.05**	**0.07**
	**Ct.Th (mm)**	**0.21 ± 0.01**	**0.19 ± 0.01**	** < 0.001**
	Perimeter (mm)	5.98 ± 0.3	6.18 ± 0.27	0.13
	D_AP_outer_ (mm)	1.38 ± 0.09	1.44 ± 0.1	0.25
	D_ML_outer_ (mm)	2.16 ± 0.11	2.24 ± 0.12	0.15
	D_AP_inner_ (mm)	0.99 ± 0.08	1.06 ± 0.1	0.1
	D_ML_inner_ (mm)	1.73 ± 0.1	1.78 ± 0.13	0.31
	AP/ML	0.64 ± 0.03	0.64 ± 0.05	0.48
	I_min_ (mm^4^)	0.21 ± 0.04	0.21 ± 0.05	0.74
	BV/TV_TB_	0.15 ± 0.05	0.11 ± 0.05	0.11
	BS/BV (mm^−1^)	49.59 ± 5.12	52.56 ± 5.63	0.23
	Tb.N (mm^−1^)	1.85 ± 0.57	1.44 ± 0.55	0.11
	Tb.Th (mm)	0.08 ± 0.01	0.07 ± 0.01	0.21
	Tb.Sp (mm)	0.26 ± 0.04	0.3 ± 0.07	0.21
Dynamic mechanical analysis (DMA)	K* (N/mm)	**196.44 ± 35.18**	**139.84 ± 24.42**	**0.001**
	**K′ (N/mm)**	**196.14 ± 35.18**	**139.67 ± 24.38**	**0.001**
	**K′′ (N/mm)**	**10.57 ± 1.3**	**6.78 ± 1.38**	** < 0.001**
	**tan δ**	**0.055 ± 0.008**	**0.049 ± 0.004**	**0.05**
Fracture	**F**_**max**_** (N)**	**22.28 ± 3.52**	**16.92 ± 5.75**	**0.03**
	d_max_ (mm)	0.24 ± 0.05	0.24 ± 0.08	0.97
	U (Nmm)	2.9 ± 0.94	2.44 ± 1.13	0.35

**Table 2:** Comparison of measured parameters between normal phosphate diet (NPD) and high phosphate diet (HPD) groups (mean ± standard deviation) in the whole bone (WB), alveolar bone (AB), and cortical bone (CB) of mandible. Significant (p < 0.05) differences are highlighted in bold

Parameters		NPD (n = 8)	HPD (n = 10)	p value
Volumetric	BV_WB_ (mm^3^)	10.16 ± 0.4	9.83 ± 1.01	0.35
	TV_WB_ (mm^3^)	14.62 ± 0.45	14.33 ± 0.92	0.4
	BV_AB_ (mm^3^)	1.05 ± 0.07	1.01 ± 0.19	0.5
Mineral Density	BMD (mgHA/cm^3^)	940.51 ± 41.76	901.01 ± 63.98	0.14
	TMC_WB_ (mgHA)	13.75 ± 0.59	12.93 ± 1.45	0.13
	TMC_AB_ (mgHA)	1.45 ± 0.11	1.34 ± 0.26	0.28
	**Mean**_**WB**_** (mgHA/cm**^**3**^**)**	**1352.33 ± 17.43**	**1314.77 ± 20.62**	** < 0.001**
	SD_WB_ (mgHA/cm^3^)	119.53 ± 6.64	111.45 ± 11.51	0.08
	Low_5WB_ (mgHA/cm^3^)	1133.24 ± 17.13	1121.37 ± 9.09	0.11
	**High**_**5WB**_** (mgHA/cm**^**3**^**)**	**1514.7 ± 26.2**	**1480.22 ± 35.64**	**0.03**
	**Mean**_**AB**_** (mgHA/cm**^**3**^**)**	**1371.06 ± 21.52**	**1331.32 ± 18.71**	**0.001**
	SD_AB_ (mgHA/cm^3^)	100.36 ± 7.1	98.88 ± 7.92	0.68
	**Low**_**5AB**_** (mgHA/cm**^**3**^**)**	**1185.2 ± 15.42**	**1160.55 ± 11.18**	**0.002**
	**High**_**5AB**_** (mgHA/cm**^**3**^**)**	**1516.92 ± 25.97**	**1482.82 ± 24.86**	**0.01**

The voxel counts were multiplied by the unit volume of the voxel. The total volume (TV_WB_) includes volumes of the whole bone (BV_WB_) and bone marrow. For a femur, volumes of cortical bone (CB) and trabecular bone (TB) were computed by separating the regions using a compartmentalizing method (Fig. 2A) [21, 22, 24]. The mandible was digitally dissected from a mental foramen having a width of 3.6 mm in the buccolingual direction, which includes all molars. Then, the whole bone (WB) voxels were isolated after removing teeth (Fig. 2B). The alveolar bone (AB) voxels surrounding the teeth were also identified within 100 μm from periodontal ligament. Mineral density parameters were measured by converting a gray level of each bone voxel to a value of tissue mineral density (TMD) using a calibration curve with known densities of commercial hydroxyapatite (HA) phantoms (1220 and 1540 mgHA/cm^3^). The mean, standard deviation (SD), and low and high (Low_5_ and High_5_) values at lower and upper 5th percentile values were obtained using a TMD frequency plot (Fig. 5A). The total mineral content (TMC) was computed by multiplying the mean TMD by BV at each region. A bone mineral density (BMD) was obtained by dividing TMC_WB_ by TV_WB_. The nomenclature and analysis of morphological parameters followed the guidelines for micro-CT–based assessment as suggested in a previous study [25]. CB morphological parameters were computed at a region centered at 55% (CB_55_) of the femoral length from the head, at which 3-point bending load was applied. The parameters of cortical bone area (Ct.Ar) and total area (Tt.Ar), cortical thickness (Ct.Th), periosteal periqmeter (Perimeter55), outer and inner diameters of anterior–posterior axis and medial–lateral axis (D_AP_outer_, D_AP_inner_, D_ML_outer_, and D_ML_inner_), outer diameter ratio (AP/ML), and minimum inertia (I_min_) were calculated. TB morphological parameters were determined using a cubic region of interest (0.72 × 0.72 × 1.5 mm^3^) above growth plates at the distal condyle of femur. TB bone fraction (BV/TV_TB_), surface-to-volume ratio (BS/BV), number (Tb.N), thickness (Tb.Th), and separation (Tb.Sp) were measured using a morphological software provided by the micro-CT company (CTAn, Bruker, Kontich, Belgium).

### Dynamic Mechanical Analysis (DMA) and Static Fracture Testing

**Fig. 3:**
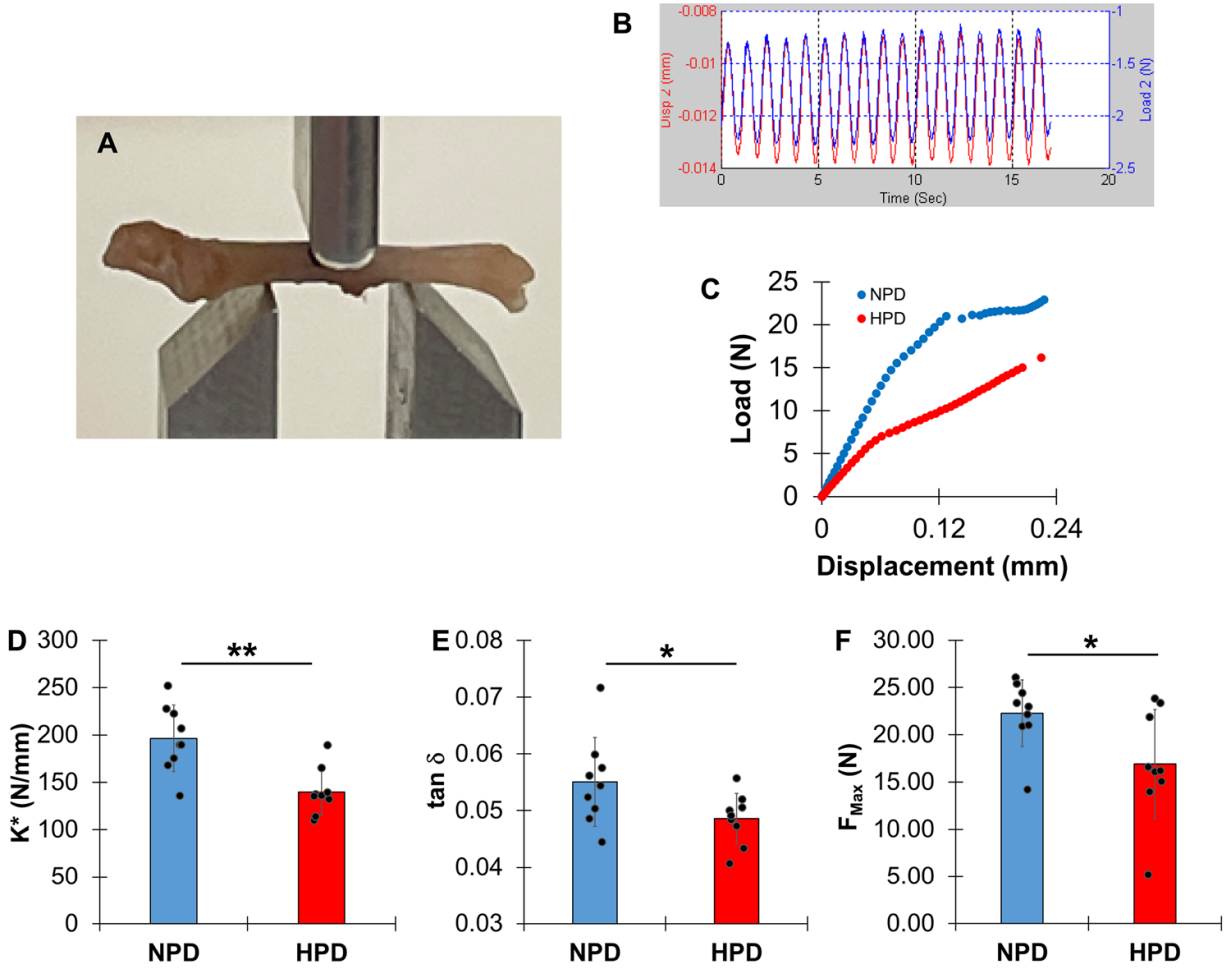
High phosphate diet decreases mechanical properties of femurs. **A** 3-point bending was applied at 55% of length from the femoral head to conduct **B** dynamic mechanical analysis (DMA) at cyclic bending displacement (-0.01 ± 0.005 mm), and finally, **C** static fracture of femurs from NPD- and HPD-fed mice. All of these non-destructive DMA, and fracture testing were performed using the same specimen. Data distribution of significantly different mechanical parameters from NPD- and HPD-fed mice including **D** dynamic stiffness (K*), **E** energy dissipation (tan δ), and **F** strength (F_MAX_). n = 9, *; p < 0.05, **; p < 0.001

Following non-destructive micro-CT scanning, the femurs were compressed at 55% length of the femur from the femoral head in the anterior–posterior direction using a 3-point bending jig with 5 mm span length (Fig. 3A). Phosphate buffered saline (PBS)-soaked paper towels were used to wet the specimens for the entire period of experiment. Dynamic mechanical testing followed the previous studies using an electromagnetic loading machine (ELF 3230, Bose Corporation, Framingham, MA, USA) [22, 26, 27]. Dynamic mechanical analysis (DMA) used non-destructive bending oscillatory displacements (0.01 ± 0.005 mm) at 0.5, 1, 2, and 3 Hz using (Fig. 3B). Dynamic complex (K*), elastic (storage) (K′), and viscous (loss) (K′′) stiffness, and tangent delta (tan δ) (K′′/K′) were assessed. The tan δ was used to measure energy dissipation ability, and the DMA values at each frequency were averaged. Static fracture testing was performed after non-destructive DMA testing. The femurs (n = 9 for NP and n = 8 for HP) were subjected to compressive bending at a displacement rate of 0.5 mm/second. Maximum force (F_max_), displacement (d_max_), and toughness (U) were assessed at fracture using the load–displacement curve (Fig. 3C).

### Histological Analysis

Dissected tibiae were fixed in 10% neutral buffered formalin (StatLab Medical Product) for 24 h at 4 °C (n = 5 per group). Subsequently, tibiae were demineralized in ethylenediaminetetraacetic acid (pH 7.3, Gojira Fine Chemicals, LLC) at 4 °C for 28 days. The demineralized tibiae were then embedded in paraffin and sectioned into 5-μm-thick slices using a microtome. The sections were stained for histomorphometric analysis using tartrate-resistant acid phosphatase (TRAP) (FUJIFILM Wako Pure Chemical Co.) following the manufacturer’s protocols. Immunohistochemical staining was employed to detect osteoblasts. Paraffin sections were rehydrated, treated with 3.0% hydrogen peroxide, and blocked following the instructions of an avidin/biotin-based peroxidase immunostaining kit (Vector Laboratories). Sections were then incubated overnight at 4 °C with primary antibodies: rabbit polyclonal anti-Runx2 (1:200 dilution, ab23981; Abcam) for osteoblasts. Secondary antibodies, goat anti-rabbit IgG (1:1000 dilution, ab6721; Abcam) conjugated with horseradish peroxidase (HRP), were used for detection. Protein expression was visualized using a peroxidase substrate solution, and a final counterstain with Harris hematoxylin (Wako Pure Chemical Industries, Ltd.) was applied. Areas of interest (AOIs) were defined as regions up to 200 μm from the growth plate and included primary spongiosa and cortical bone. The stained sections were examined and photographed under light microscopy. In these AOIs, osteoclast numbers per bone perimeter (N.Oc/BS) and the density of Runx2 + osteoblasts were calculated semi-automatically.

### Statistical Analysis

The Shapiro–Wilk test was performed to confirm normality of data for each parameter of NP and HP groups. If the data normally distributed, a Student’s t-test was performed to compare each parameter between groups. If the distribution of data was not normal, Mann–Whitney U test was used to compare data from NP and HP groups. Two outliers outside of the 95% confidential limit were excluded from the TMD parameters in the mandible. Mechanical testing data from a femur in NP and HP group was not obtained due to an experimental error. A stepwise regression was performed to identify key parameters significantly associated with strength (maximum force, F_max_), using elastic (storage) stiffness (K′), volumetric, mineral, and morphological parameters as predictive variables for each group. Significance was set at p < 0.05.

## Results

### High Phosphate Diet Increases Serum Phosphate Levels Without Affecting Body Weight and Body Composition

We recently demonstrated that twelve weeks of high phosphate (HP) diet decreases exercise capacity by altering skeletal muscle metabolism [28]. Because phosphate is a main mineral component of bone and there are overlapping mechanisms regulating mechanotransduction in muscle and bone [29, 30], we specifically investigated structural and mechanical behavior of bone in the same dietary model system. Wild type male mice were fed normal phosphate diet (NPD) or high phosphate diet (HPD) for 18 weeks. We did not observe differences in body weight gain between NPD and HPD throughout the dietary study, nor did we observe changes in lean and fat mass after 17 weeks of HPD compared to NPD (Fig. 1A-B). Serum phosphate levels were significantly increased in mice fed HPD within a physiological non-pathological range (p < 0.001), while serum calcium, serum glucose, and serum creatinine were unaffected (Fig. 1C–F). Serum fibroblast growth factor 23 (FGF23) levels were also increased in mice fed HPD (p < 0.02), with concurrent increases in serum parathyroid hormone (PTH) (p < 0.002) both within physiological non-pathological ranges (Fig. 1G–H). Serum Collagen Type I C-Telopeptide (CTX-I) (p < 0.01) and serum Procollagen Type 1 Intact N-terminal Propeptide (P1NP) (p < 0.001) were both increased in the HPD group (Fig. 1I–J). Interestingly, the ratio of P1NP to CTX-I was not significantly different in mice fed NPD or HPD (Fig. 1K) suggesting equivalent augmentation of bone resorption and formation by HPD, which could lead to increased, but balanced, bone turnover.

### High Phosphate Diet Alters Cellular Composition of Bone

**Fig. 4:**
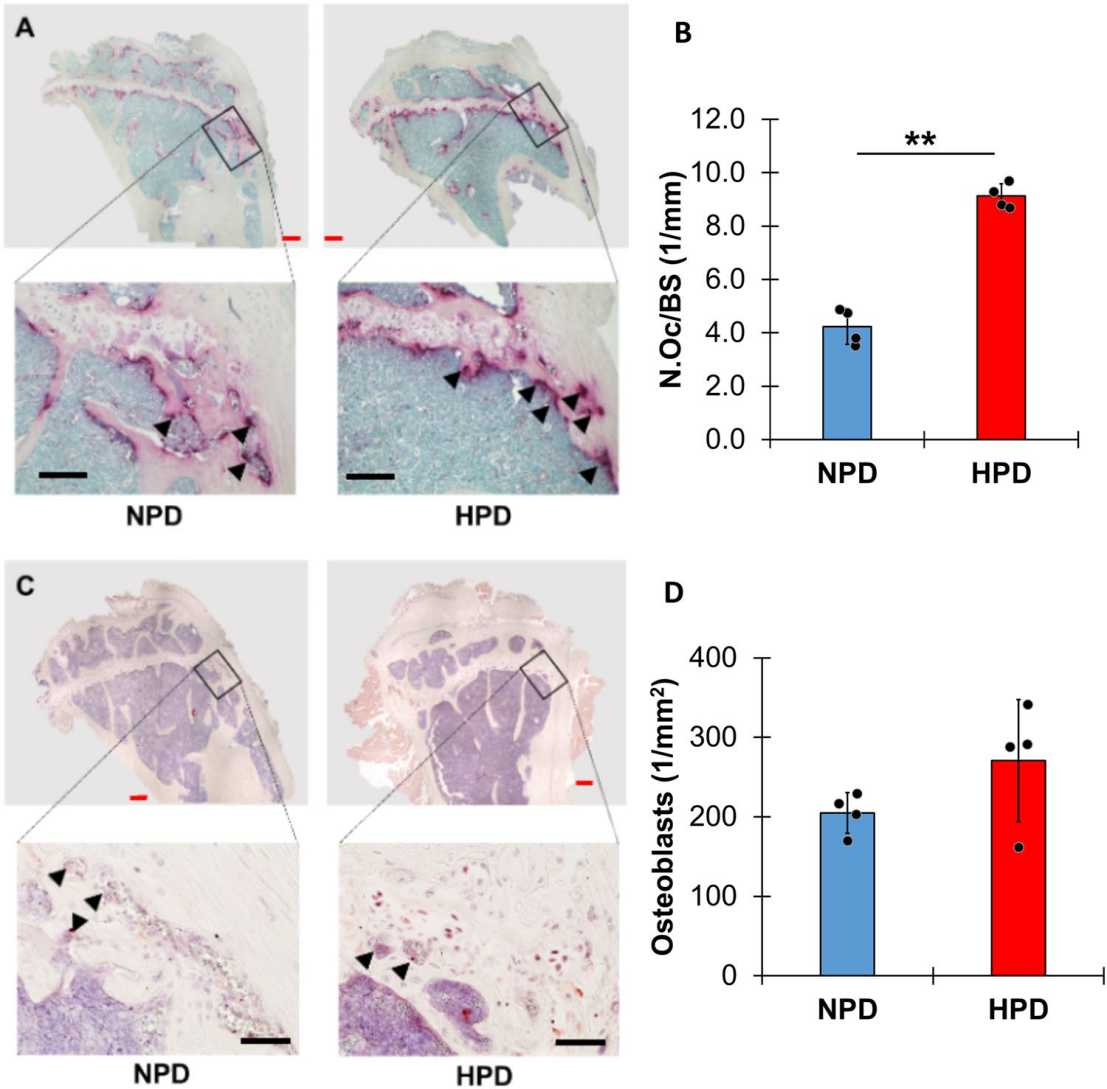
High phosphate diet alters cellular composition of bone. **A** Examples of tartrate-resistant acid phosphatase (TRAP) staining on paraffin sections of tibias from NPD- and HPD- fed mice, and **B** quantification of TRAP positive cells (the number of osteoclasts (N.Oc) and bone surface (BS)). **C** Examples of Runt-related transcription factor 2 (Runx2) staining in tibias from NPD- and HPD-fed mice, and **D** quantification of Runx2 positive cells (osteoblasts). Upper panels in A and C were visualized with a 5X objective; red scale bar = 200 μm. Lower panels were visualized with a 20X objective; black scale bar = 100 μm

To gain mechanistic insight into bone characteristics altered with HPD, we performed histological analysis on tibias from mice fed HPD or NPD for 18 weeks. To determine whether HPD altered abundance of osteoclasts we performed tartrate-resistant acid phosphatase (TRAP) staining on paraffin sections of tibias. Quantification of TRAP-positive cells within 200 µm of the growth plate indicates a significant increase in the number of osteoclasts in tibias from HPD- compared to NPD-fed mice (Fig. 4A–B). We also quantified osteoblasts by staining for runt-related transcription factor 2 (Runx2) in tibias from NPD- and HPD-fed mice. The density of Runx2 + osteoblasts showed a tendency to increase in tibias from mice fed HPD compared to the NPD (Fig. 4C–D). Collectively, these data demonstrate that HPD alters the cellular composition of bone, consistent with our findings of increased osteoclast and osteoblast activity in the serum.

### High Phosphate Diet Decreases Tissue Mineral Density (TMD), Cortical Bone Thickness (Ct.Th), and Mechanical Properties Without Affecting Bone Volume and Other Morphology

**Fig. 5:**
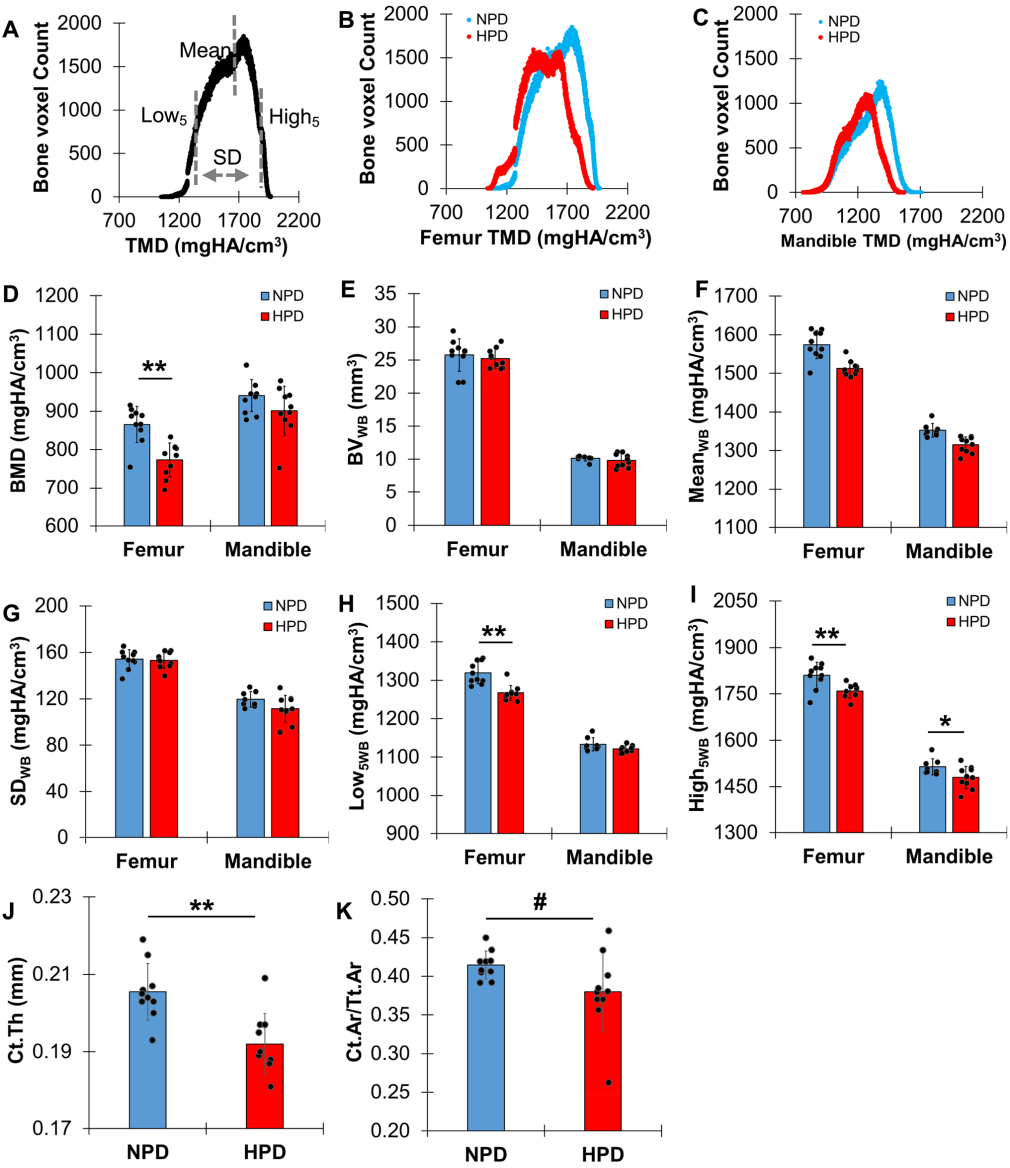
High phosphate diet decreases bone characteristics of femurs and mandibles. **A** Tissue mineral density (TMD) parameters were determined using an individual frequency plot of each region, a typical TMD frequency plot of WB in **B** femur and **C** mandible from NPD- and HPD-fed mice. Comparisons with data distribution of key parameters between NPD and HPD (n = 10 for each group of femur, n = 8 for NPD and n = 10 for HPD mandible) including **D** bone mineral density (BMD), **E** bone volume (BV_WB_), **F** mean tissue mineral density (TMD) (Mean_WB_), **G** heterogeneity of TMD (SD_WB_), **H** lower and **I** upper 5th percentile values of TMD (Low_5WB_ and High_5WB_) of WB, **J** cortical bone thickness (Ct.Th), and **K** a ratio of cortical bone area to total area at the 55% of length from the femoral head. *; p < 0.05, **; p < 0.01, #; p = 0.07

Each region of femur and mandible was successfully isolated using the digital compartmentation procedure for the 3D micro-CT image (Fig. 2). For both femur and mandible, the values of volumetric and morphology parameters were not significantly different between NPD and HPD-fed mice (p > 0.08) (Table 1,2, and Fig. 5E). These results indicate that overall bone content is maintained with HPD and are consistent with the P1NP and CTX-I assays that suggested increased but balanced bone turnover, leading to no changes in bone volume. In contrast, mineral density was altered with HPD in both femur and mandible. Femurs from HPD-fed mice showed significant decreases in bone mineral density (BMD), and mean (Mean), low and high (Low_5_ and High_5_) values of the tissue mineral density (TMD) (Table 1 and Fig. 5A–D, F, H–I). The Low_5_ and High_5_ values represent the 5% lowest value and the 5% highest value of TMD, respectively (Fig. 5A), and reflect newly formed, poorly mineralized bone (Low_5_) and mature, highly mineralized bone (High_5_) [12, 21]. As such, decreases in both these values indicate that more newly formed bone is produced (increased osteoblast activity), while mature bone is lost at a higher rate (increased osteoclast activity). These results are also consistent with the serum P1NP and CTX-I values (Fig. 1). Femurs from HPD-fed mice also showed decreased cortical bone thickness (Ct.Th) compared to the NPD-fed mice (Table 1 and Fig. 4J). Mandibles from HPD-fed mice showed significant decreases in Mean and High_5_ values of TMD relative to NPD-fed mice for whole bone (Table 2 and Fig. 5F, I). In alveolar bone, mandibles demonstrated decreased Mean, Low_5_, and High_5_ values of the TMD (Table 2) in response to HPD. Thus, these data along with the serum analyses indicate that HPD produced similar effects on mineralization of both femur and mandible by increasing turnover through enhanced osteoblast and osteoclast activity. As mineral density is a key regulator of bone mechanical properties, dynamic mechanical analysis (DMA) was performed on femurs. HPD significantly decreased femur dynamic stiffness (K*), energy dissipation (tan δ), and maximum force (F_max_) compared to NPD (p < 0.05) (Table 1 and Fig. 3), indicating diminished mechanical properties.

The stepwise regression analysis indicates that, for the NPD group, K′ was the only significant parameter explaining F_max_ (r^2^ = 0.75, p = 0.003), while other parameters did not improve the regression model. Further stepwise regression on K′ identified Ct.Th, BMD, and AP/ML as predictive variables for building a significant model (r^2^ = 0.93, p = 0.002). For the HPD group, F_max_ was predicted by Low_5CB_, K′, and Ct.Th (r^2^ = 0.99, p < 0.001), and K′ was explained by BMD and BV_CB_ (r^2^ = 0.79, p = 0.01).

## Discussion

Food preservatives and flavor enhancers containing excess phosphate are utilized in processed foods that are frequently consumed by adults, and the consequences of increased phosphate intake are not entirely understood. In this study we investigate the effects of high phosphate diet (HPD) on mouse mandibles, femurs, and tibias. Male mice were fed an HPD containing proportional increases in phosphate to reflect the increased consumption of 2.5 times the EAR and twice the RDA of phosphate by adults in the U.S [3]. Eighteen-week HPD decreased bone mineral density (BMD) and mechanical properties of mouse femurs, consistent with the previous results in female mice fed acute HPD [7] and female and male mice fed chronic HPD [8, 9]. The current study also provides more detailed analysis of the HPD-associated alterations of mouse femurs, mandibles, and tibias, showing that HPD caused increased but balanced osteoblast and osteoclast activity, leading to loss of bone quality (TMD and thickness) without changing bone quantity (TMC and volume). As a result, HPD exacerbated mechanical properties of the femur, increasing the risk of fracture, which could be due to changes in cellular composition of bone.

Micro-CT based TMD measurements have been widely used for rodent models because its high resolution can provide detailed information of bone properties [25]. However, most micro-CT-based analyses measure BMD as a bone quantity by inaccurately assuming that TMD is homogeneous in bone, which doesn’t account for local dynamic bone turnover (modeling and remodeling) [14, 17]. In addition to bone quantity, the current study investigated bone quality including the heterogeneous distribution of TMD and morphology, which can alter mechanical characteristics of bone. The current study demonstrates that HPD stimulated increased bone turnover from augmented but balanced osteoblast and osteoclast activity, resulting in diminished mineralization properties and mechanical strength, without changing overall bone volume.

For both femur and mandible, we found that the TMD values (Mean, Low_5_, High_5_) shifted to lower values in response to HPD-, compared to NPD-fed mice. Decreases in Low_5_ and High_5_ values indicate that more newly formed, less mineralized bone tissues are added, and more mineralized existing bone tissues are removed resulting in the reduction of mean TMD value. As such, the TMD distribution represents a fingerprint of the mineralization process in bone [16, 17]. Analyses of TMD distribution suggest that HPD triggers active bone resorption followed by bone formation, leading to the significantly lower mean of TMD. These results are also consistent with our findings of enhanced but balanced osteoclast and osteoblast numbers and activities with HPD.

The alveolar bone (AB) region surrounding teeth also sustains active bone modeling and remodeling continuously stimulated by direct transmission of dynamic chewing force. HPD decreased the mean TMD_AB_ by reduction of Low_5AB_ and High_5AB_ as found in the TB region of femur. In contrast to the femur, Low_5AB_ values of the whole bone (WB) region of mandible were not significantly different between HPD- and NPD-fed mice. Previous studies reported a greater ability of bone mineralization by bone marrow stem cells (BMSCs) in jawbone than in orthopedic bone, likely due to their different embryonic origins [12, 13]. These findings suggest that the newly formed bone tissue in mandible mineralizes faster than the femur. Consequently, the mandible may be impacted to a lesser degree than the femur in response to HPD.

The results of serum analysis provide further support that HPD triggers active bone turnover. Both PTH and FGF23, which are recognized to become elevated in the presence of high serum phosphate levels, were increased by HPD. It is well established that chronically high levels of PTH increase bone turnover [31]. In the current model, HPD increased levels of the osteoclastic resorption marker CTX-I and the bone formation marker P1NP in serum along with cell numbers. The ratio of P1NP to CTX-I was not significantly different between HPD- and NPD-fed mice (Fig. 1K), indicating that even though bone resorption and formation were enhanced by HPD, the balance between these activities were maintained, leading to no change in the overall volume of bone. However, the thickness of cortical bone (Ct.Th) was significantly reduced with HPD. Although not significantly different, the perimeter, inner and outer diameters within the femur are larger with HPD- compared to the NPD-fed mice. While bone formation develops on the periosteum, both bone resorption and formation are observed on the endosteum of mouse femur [32]. In the osteoporotic femur, active bone remodeling causes endocortical resorption and periosteal apposition of bone leading to rapid cortical thinning [33]. The bone volume can be maintained by compromising cortical thinning with increasing diameter of the femur. The phenomenon was observed in the HPD-fed mice: the cortical area (Ct.Ar) to total area (Tt.Ar) was smaller due to increasing Tt.Ar by larger diameters with HPD, while Ct.Ar was identical to those with NPD.

As the mechanical properties of mouse femur were assessed using the bending test at the cortical bone, lower TMD values and thinning Ct.Th contributed to lower dynamic stiffness (K^*^) and femoral strength (F_max_) in response to HPD, compared to NPD. All other morphological parameters that may determine the bending properties of femurs (Ct.Ar, Tt.Ar, Perimeter, diameters, AP/ML, and I_min_) [34, 35] were not changed. These findings are in agreement with the notion that newly formed bone tissues have less mineral content, giving rise to weak mechanical properties of bone tissue [14, 36, 37]. Many studies observed that increased microdamages can develop in a weak bone under physiological levels of loading [38–41]. The non-destructive dynamic mechanical characteristics of bone account for its stability to bear fatigue loading during daily activity. HPD significantly reduced the ability of viscoelastic energy dissipation (tan δ) in bone, which may progressively lead to the development of microdamages in a local region of the femur under long-term weight-bearing cyclic loading.

On the other hand, the results of stepwise regression analysis that K′ plays a central role in determining femoral strength in both groups. Additionally, reductions in tissue mineral density in newly formed bone (Low_5CB_) and in Ct.Th contributed to reduced femoral strength in the HPD group. BMD appears to be an important determinant of elastic stiffness in both groups. BMD is determined as the total tissue mineral density (TMD) within the whole bone volume (TV_WB_). While TV_WB_ remained unchanged, HPD led to a reduction in TMD, suggesting that elastic stiffness was compromised due to altered mineralization. Taken together, these findings give an insight that HPD aggravates femoral strength by reducing the quality (TMD and Ct.Th) of the femur, even though its quantity (TMC and volume) remains unchanged.

## Limitations

The current study provides new insight into the mechanical properties characterizing the impact of HPD on bone health. Several HPD mouse studies have reported only some of the same effects of HPD on bone characteristics compared to the current 18-week HPD study initiated in mature adult males aged 20 weeks, when bones are fully formed. These differences, and limitations to the study, could be due to age differences at the start of the study [42], sexual dimorphism of hormonal regulation which impacts bone structure and mechanical properties [43, 44], the composition and/or length of the diet, or a combination of all variables. For example, a study initiated in 10-week-old female mice, when bones are still maturing, reported that 20-week 2% HPD decreased femur cortical thickness, increased serum phosphate, serum FGF23, and serum PTH, but reported no differences in CTX or P1NP [45]. In contrast, 4-week 2% HPD in mature adult male mice decreased cortical BMD independent of PTH, but other serum parameters were not reported. The same study reported that 58-week 1.6% HPD decreased cortical and total BMD and increased serum phosphate and PTH levels, but did not impair kidney function [9]. A limitation of the current study is the limited investigation on kidney function and additional histological studies to directly investigate whether HPD can develop microdamage in bone.

Another limitation of this study is that a potential effect of muscle activity altered by HPD on the bone was not investigated. Previous studies indicated that HPD alters metabolic and functional characteristics of muscle [28]. As muscles attached on the bone surface transmit force to stimulate bone cells, HPD may also have an indirect effect on bone by controlling muscle behavior. Direct associations between alterations of muscle and bone characteristics remain to be investigated.

Another limitation of this study is that we have not performed additional experiments to determine whether the effects of HPD in our mouse cohorts were reversible upon return to normal diets. A very recent study showed that mice fed HPD from the ages of 10 to 20 weeks and returned to NPD for another 10 weeks did not show improvements in the diminished bone quality caused by HPD [46]. In this previous study, mice were started on HPD when they had not yet reached peak bone mass (which occurs typically at 4 to 5 months of age), so it may be possible that high phosphate levels interfere with accumulation of this peak mass. In contrast, our HPD diet was administered from 20 to 38 weeks of age when the skeletons were fully mature, so it is unclear how the bone quality of the adult mice in our study may be affected.

Complete understanding of the physiological and pathological effects HPD on bone health is still lacking. Although we have a general understanding of the temporal effects of HPD on bone health, other organs have not been investigated. There is also some evidence that a low phosphate diet (LPD) is beneficial, and switching from HPD to LPD may preserve bone health [45]. Future studies will expand on this preliminary report to determine whether normalizing dietary phosphate intake reverses the negative effects of HPD to improve overall muscle and bone health in the long run.

## Conclusion

In conclusion, HPD likely stimulates active bone remodeling that couples increased osteoclastic bone resorption and osteoblastic bone formation of both femur and mandible. As a result, the tissue mineral density (TMD) parameters decreased with HPD and increased bone turnover to remove more mineralized existing bone tissues and deposit less mineralized new bone tissues. Bone mineral density (BMD) after HPD decreased because the quality (TMD and Ct.Th) of femur decreased while the quantity (TMC and volume) of bone was not changed in either femur or mandible. These alterations of bone in response to HPD also aggravates mechanical characteristics of the femur, which increases the risk of fracture. The current findings provide detailed information of how excessive dietary phosphate substantially alters characteristics of bone and weakens the load-bearing capacity. A better understanding of how the HPD influences the balance of bone remodeling is needed to prevent bone fracture.

## References

[1] Sullivan CM, Leon JB, Sehgal AR (2007) Phosphorus-containing food additives and the accuracy of nutrient databases: implications for renal patients. J Ren Nutr 17(5):350–354 17720105 10.1053/j.jrn.2007.05.008 PMC2020846

[2] Lee AW, Cho SS (2015) Association between phosphorus intake and bone health in the NHANES population. Nutr J 14:28 25856461 10.1186/s12937-015-0017-0 PMC4389665

[3] Vorland CJ, Stremke ER, Moorthi RN, Hill Gallant KM (2017) Effects of excessive dietary phosphorus intake on bone health. Curr Osteoporos Rep 15(5):473–482 28840444 10.1007/s11914-017-0398-4 PMC5693714

[4] Chang AR, Lazo M, Appel LJ, Gutierrez OM, Grams ME (2014) High dietary phosphorus intake is associated with all-cause mortality: results from NHANES III. Am J Clin Nutr 99(2):320–327 24225358 10.3945/ajcn.113.073148 PMC3893724

[5] Bergwitz C, Juppner H (2011) Phosphate sensing. Adv Chronic Kidney Dis 18(2):132–144 21406298 10.1053/j.ackd.2011.01.004 PMC3059779

[6] Campos-Obando N, Koek WNH, Hooker ER, van der Eerden BC, Pols HA, Hofman A, van Leeuwen JP, Uitterlinden AG, Nielson CM, Zillikens MC (2017) Serum phosphate is associated with fracture risk: the Rotterdam study and MrOS. J Bone Miner Res 32(6):1182–1193 28177140 10.1002/jbmr.3094 PMC5466477

[7] Koyama Y, Rittling SR, Tsuji K, Hino K, Salincarnboriboon R, Yano T, Taketani Y, Nifuji A, Denhardt DT, Noda M (2006) Osteopontin deficiency suppresses high phosphate load-induced bone loss via specific modulation of osteoclasts. Endocrinology 147(6):3040–3049 16513836 10.1210/en.2005-0671

[8] Krishnarao GV, Draper HH (1972) Influence of dietary phosphate on bone resorption in senescent mice. J Nutr 102(9):1143–1145 5057200 10.1093/jn/102.9.1143

[9] Ugrica M, Bettoni C, Bourgeois S, Daryadel A, Pastor-Arroyo EM, Gehring N, Hernando N, Wagner CA, Rubio-Aliaga I (2021) A chronic high phosphate intake in mice is detrimental for bone health without major renal alterations. Nephrol Dial Transplant. 10.1093/ndt/gfab015 33515264 10.1093/ndt/gfab015

[10] Guimarães LM, Valeriano AT, Rebelo Pontes HA, Gomez RS, Gomes CC (2022) Manifestations of hyperparathyroidism in the jaws: concepts, mechanisms, and clinical aspects. Oral Surg Oral Med Oral Pathol Oral Radiol 133(5):547–555 35181256 10.1016/j.oooo.2021.08.020

[11] Luo E, Liu H, Zhao Q, Shi B, Chen Q (2019) Dental-craniofacial manifestation and treatment of rare diseases. Int J Oral Sci 11(1):9 30783081 10.1038/s41368-018-0041-y PMC6381182

[12] Liu J, Watanabe K, Dabdoub SM, Lee BS, Kim DG (2022) Site-specific characteristics of bone and progenitor cells in control and ovariectomized rats. Bone 163:116501 35872108 10.1016/j.bone.2022.116501

[13] Akintoye SO, Lam T, Shi S, Brahim J, Collins MT, Robey PG (2006) Skeletal site-specific characterization of orofacial and iliac crest human bone marrow stromal cells in same individuals. Bone 38(6):758–768 16403496 10.1016/j.bone.2005.10.027

[14] Liu J, Kim EK, Ni A, Kim YR, Zheng F, Lee BS, Kim DG (2021) Multiscale characterization of ovariectomized rat femur. J Biomech 122:110462 33915473 10.1016/j.jbiomech.2021.110462 PMC8166321

[15] Hernandez CJ, Keaveny TM (2006) A biomechanical perspective on bone quality. Bone 39(6):1173–1181 16876493 10.1016/j.bone.2006.06.001 PMC1876764

[16] Roschger P, Paschalis EP, Fratzl P, Klaushofer K (2008) Bone mineralization density distribution in health and disease. Bone 42(3):456–466 18096457 10.1016/j.bone.2007.10.021

[17] Ruffoni D, Fratzl P, Roschger P, Klaushofer K, Weinkamer R (2007) The bone mineralization density distribution as a fingerprint of the mineralization process. Bone 40(5):1308–1319 17337263 10.1016/j.bone.2007.01.012

[18] Czaya B, Heitman K, Campos I, Yanucil C, Kentrup D, Westbrook D, Gutierrez O, Babitt JL, Jung G, Salusky IB, Hanudel M, Faul C (2022) Hyperphosphatemia increases inflammation to exacerbate anemia and skeletal muscle wasting independently of FGF23-FGFR4 signaling. Elife 11:e74782 35302487 10.7554/eLife.74782 PMC8963881

[19] Maginot M, Lin S, Liu Y, Yuan B, Feng JQ, Aswath PB (2015) The in vivo role of DMP-1 and serum phosphate on bone mineral composition. Bone 81:602–613 26303287 10.1016/j.bone.2015.08.018

[20] Feng JQ, Ward LM, Liu S, Lu Y, Xie Y, Yuan B, Yu X, Rauch F, Davis SI, Zhang S, Rios H, Drezner MK, Quarles LD, Bonewald LF, White KE (2006) Loss of DMP1 causes rickets and osteomalacia and identifies a role for osteocytes in mineral metabolism. Nat Genet 38(11):1310–1315 17033621 10.1038/ng1905 PMC1839871

[21] Kim DG, Navalgund AR, Tee BC, Noble GJ, Hart RT, Lee HR (2012) Increased variability of bone tissue mineral density resulting from estrogen deficiency influences creep behavior in a rat vertebral body. Bone 51(5):868–875 22944606 10.1016/j.bone.2012.08.124 PMC3455132

[22] Kim DG, Jeong YH, McMichael BK, Bahler M, Bodnyk K, Sedlar R, Lee BS (2018) Relationships of bone characteristics in MYO9B deficient femurs. J Mech Behav Biomed Mater 84:99–107 29754047 10.1016/j.jmbbm.2018.05.003

[23] Zauel R, Yeni YN, Christopherson GT, Cody DD, Fyhrie DP (2004) Segmentation algorithm for accurate 3D representation of microcomputed tomographic images of human vertebral bodies. Trans. of Orthopaedic Res. Society 29:1018

[24] Buie HR, Campbell GM, Klinck RJ, MacNeil JA, Boyd SK (2007) Automatic segmentation of cortical and trabecular compartments based on a dual threshold technique for in vivo micro-CT bone analysis. Bone 41(4):505–515 17693147 10.1016/j.bone.2007.07.007

[25] Bouxsein ML, Boyd SK, Christiansen BA, Guldberg RE, Jepsen KJ, Muller R (2010) Guidelines for assessment of bone microstructure in rodents using micro-computed tomography. J Bone Miner Res 25(7):1468–1486 20533309 10.1002/jbmr.141

[26] Kim DG, Haghighi A, Kwon HJ, Coogan JS, Nicolella DP, Johnson TB, Kim HD, Kim N, Agnew AM (2017) Sex dependent mechanical properties of the human mandibular condyle. J Mech Behav Biomed Mater 71:184–191 28342326 10.1016/j.jmbbm.2017.03.012

[27] Kim DG, Kwon HJ, Jeong YH, Chien HH, Crance S, Agnew AM, Battula S, Lee JW, Wen HB (2016) Associations of resonance frequency analysis with dynamic mechanical analysis of dental implant systems. Clin Implant Dent Relat Res 18(2):332–341 25810026 10.1111/cid.12319

[28] Peri-Okonny P, Baskin KK, Iwamoto G, Mitchell JH, Smith SA, Kim HK, Szweda LI, Bassel-Duby R, Fujikawa T, Castorena CM, Richardson J, Shelton JM, Ayers C, Berry JD, Malladi VS, Hu MC, Moe OW, Scherer PE, Vongpatanasin W (2019) High-phosphate diet induces exercise intolerance and impairs fatty acid metabolism in mice. Circulation 139(11):1422–1434 30612451 10.1161/CIRCULATIONAHA.118.037550 PMC6411426

[29] Alfieri R, Vassalli M, Viti F (2019) Flow-induced mechanotransduction in skeletal cells. Biophys Rev 11(5):729–743 31529361 10.1007/s12551-019-00596-1 PMC6815304

[30] Goodman CA, Hornberger TA, Robling AG (2015) Bone and skeletal muscle: key players in mechanotransduction and potential overlapping mechanisms. Bone 80:24–36 26453495 10.1016/j.bone.2015.04.014 PMC4600534

[31] Wein MN, Kronenberg HM (2018) Regulation of bone remodeling by parathyroid hormone. Cold Spring Harb Perspect Med. 10.1101/cshperspect.a031237 29358318 10.1101/cshperspect.a031237 PMC6071549

[32] Scheuren A, Wehrle E, Flohr F, Müller R (2017) Bone mechanobiology in mice: toward single-cell in vivo mechanomics. Biomech Model Mechanobiol 16(6):2017–2034 28735414 10.1007/s10237-017-0935-1

[33] Eastell R, O’Neill TW, Hofbauer LC, Langdahl B, Reid IR, Gold DT, Cummings SR (2016) Postmenopausal osteoporosis. Nat Rev Dis Primers 2:16069 27681935 10.1038/nrdp.2016.69

[34] Jepsen KJ, Silva MJ, Vashishth D, Guo XE, van der Meulen MC (2015) Establishing biomechanical mechanisms in mouse models: practical guidelines for systematically evaluating phenotypic changes in the diaphyses of long bones. J Bone Miner Res 30(6):951–966 25917136 10.1002/jbmr.2539 PMC4794979

[35] Voide R, van Lenthe GH, Muller R (2008) Bone morphometry strongly predicts cortical bone stiffness and strength, but not toughness, in inbred mouse models of high and low bone mass. J Bone Miner Res 23(8):1194–1203 18348694 10.1359/jbmr.080311

[36] Mulder L, Koolstra JH, den Toonder JMJ, van Eijden TMGJ (2007) Intratrabecular distribution of tissues stiffness and mineralization in developing trabecular bone. Bone 4(12):56–265 10.1016/j.bone.2007.04.188 17567548

[37] Kim DG, Huja SS, Lee HR, Tee BC, Hueni S (2010) Relationships of viscosity with contact hardness and modulus of bone matrix measured by nanoindentation. J Biomech Eng 132(2):024502 20370248 10.1115/1.4000936

[38] Pattin CA, Caler WE, Carter DR (1996) Cyclic mechanical property degradation during fatigue loading of cortical bone. J Biomech 29(1):69–79 8839019 10.1016/0021-9290(94)00156-1

[39] Seref-Ferlengez Z, Kennedy OD, Schaffler MB (2015) Bone microdamage, remodeling and bone fragility: how much damage is too much damage? Bonekey Rep 4:644 25848533 10.1038/bonekey.2015.11 PMC4371415

[40] Schaffler MB, Choi K, Milgrom C (1995) Aging and matrix microdamage accumulation in human compact bone. Bone 17(6):521–525 8835305 10.1016/8756-3282(95)00370-3

[41] Donahue SW, Galley SA (2006) Microdamage in bone: implications for fracture, repair, remodeling, and adaptation. Crit Rev Biomed Eng 34(3):215–271 16930125 10.1615/critrevbiomedeng.v34.i3.20

[42] Halloran BP, Ferguson VL, Simske SJ, Burghardt A, Venton LL, Majumdar S (2002) Changes in bone structure and mass with advancing age in the male C57BL/6J mouse. J of Bone and Mineral 17(6):1044–1050 10.1359/jbmr.2002.17.6.1044 12054159

[43] Wang Y, Sakata T, Elalieh HZ, Munson SJ, Burghardt A, Majumdar S, Halloran BP, Bikle DD (2006) Gender differences in the response of CD-1 mouse bone to parathyroid hormone: potential role of IGF-I. J Endocrinol 189(2):279–287 16648295 10.1677/joe.1.06351 PMC10745196

[44] Yao X, Carleton SM, Kettle AD, Melander J, Phillips CL, Wang Y (2013) Gender-dependence of bone structure and properties in adult osteogenesis imperfecta murine model. Ann Biomed Eng 41(6):1139–1149 23536112 10.1007/s10439-013-0793-7 PMC3703620

[45] Roberts JL, Yu M, Viggeswarapu M, Arnst JL, Pacifici R, Beck GR Jr. (2023) Dietary phosphorus consumption alters T cell populations, cytokine production, and bone volume in mice. JCI Insight. 10.1172/jci.insight.154729 37079375 10.1172/jci.insight.154729 PMC10322696

[46] Arnst JL, Alappan UD, Viggeswarapu M, Beck GR Jr (2025) Sustained and reversible effects of a dietary phosphate intake on bone and mineral metabolism during aging. Geroscience. 10.1007/s11357-025-01714-6 40455097 10.1007/s11357-025-01714-6 PMC12972357

